# Bioactive peptide isolated from sesame seeds inhibits cell proliferation and induces apoptosis and autophagy in leukemic cells

**DOI:** 10.17179/excli2021-3406

**Published:** 2021-03-23

**Authors:** Kamolchanok Deesrisak, Yodying Yingchutrakul, Sucheewin Krobthong, Sittiruk Roytrakul, Chawalit Chatupheeraphat, Paweena Subkorn, Usanarat Anurathapan, Dalina Tanyong

**Affiliations:** 1 Department of Clinical Microscopy, Faculty of Medical Technology, Mahidol University, Nakhon Pathom 73170, Thailand; 2 Proteomics Research Team, National Omics Center, National Science and Technology Development Agency, Pathum Thani 12120, Thailand; 3 Functional Proteomics Technology Laboratory, Functional Ingredients and FoodInnovation Research Group, National Center for Genetic Engineering and Biotechnology, National Science and Technology for Development Agency, Pathum Thani 12120,Thailand; 4 Department of Pediatrics, Faculty of Medicine Ramathibodi Hospital, Mahidol University, Bangkok 10400, Thailand

**Keywords:** apoptosis, autophagy, bioactive peptide, leukemia, sesame

## Abstract

Leukemia is the most common type of hematological malignancies. Several natural products including bioactive peptides have been explored and studied for their anti-leukemic activities. In the present study, anti-leukemic peptide, IGTLILM (IM-7), was isolated and identified from the protein hydrolysate of sesame seeds by reverse phase-solid phase extraction, off-gel fractionation and nano LC-MS/MS. The cytotoxic effects of IM-7 were studied in MOLT-4 and NB4 acute leukemic cell lines using an MTT assay. The induction of apoptosis and autophagy was investigated by flow cytometry using Annexin V-FITC/PI staining and anti-LC3/FITC antibodies, respectively. The mRNA alterations of apoptotic and autophagic-related genes were determined by reverse transcription-quantitative PCR. The present study found that IM-7 inhibited the proliferation of MOLT-4 and NB4 cells in dose-dependent manner, but it showed a minimal effect on healthy mononuclear cells. IM-7 activated apoptosis and autophagy through the upregulation of CASP3, ULK1 and BECN1 and the downregulation of BCL2. In addition, IM-7 enhanced the cytotoxic effect of the anti-leukemic drug, daunorubicin. The findings suggested that IM-7 was potent to suppress the proliferation of MOLT-4 and NB4 leukemic cells and induce apoptosis and autophagy through the regulation of caspase 3-Bcl-2 and ULK1-Beclin1, respectively.

## Introduction

Leukemia is one of the hematological malignancies characterized by uncontrolled production of abnormal leukocytes. Comparing to all sites of cancer, 2.4 % cases and 3.2 % deaths of leukemia were reported (Bray et al., 2018[3]). Chemotherapy is the main treatment for most types of leukemia. However, the complications from intensive chemotherapy are the limitation and may lead to relapse in some patients (Jarfelt et al., 2016[16]; Yilmaz et al., 2019[33]). Over the years, therapeutic peptides have been studied and developed as drugs due to their advantages, including small size, ease of synthesis and modification, tumor-penetrating ability and low toxicity (Thundimadathil, 2012[30]). The anti-leukemic peptides extracted from natural sources, including plants and animals, have been reported. For example, cyclotides extracted from Violaceae families and *Psychotria leptothyrsa* have been shown to possess dose-dependent cytotoxicity in U-937 cell line (Gerlach et al., 2010[11]; Herrmann et al., 2008[14]; Svangård et al., 2007[29]; Yeshak et al., 2011[32]). Moreover, cationic peptide-CM4 from hemolymph of the silkworm *Bombyx mori* has been reported to inhibit the proliferation of leukemic cells (Chen et al., 2010[6]).

Sesame (*Sesamum indicum* L.) is one of the oilseed crops commonly used in various types of food and pharmaceutical products. Sesame seeds contain the valuable nutrients and bioactive chemical agents such as sesamin, sesamol and sesaminol that have exhibited the therapeutic activities including antioxidant, anti-inflammatory and anticancer activities (Pathak et al., 2017[25]). Besides, the proteins and peptides derived from sesame seeds have been reported for the medicinal properties, including antioxidant (Liu and Chiang, 2008[19]; Lu et al., 2019[20]), anti-bacterial (Das et al., 2012[7]) and anti-hypertensive (Nakano et al., 2006[24]) activities. However, there is no report about the effect of sesame peptides on leukemia.

Since leukemia is characterized by the excessive production of blood cells, the induction of apoptosis or type I programmed cell death has been used for eliminating the number of leukemic cells (Cassier et al., 2017[4]). Likewise, autophagy as known as type II programmed cell death is an alternative approach for leukemia treatment (Zhang et al., 2013[36]). GX15-070 (obatoclax) has been reported to inhibit the proliferation of acute lymphocytic leukemia via the induction of apoptosis and autophagy (Heidari et al., 2010[13]). Same as resveratrol, the natural polyphenol found in several plants, it has been shown to induce the apoptotic and autophagic death in leukemic cells (Fan et al., 2018[9]; Zhang et al., 2018[37]).

Thus, the present study purposed to isolate the anti-leukemic peptides from sesame seeds and examine the underlying mechanisms related to apoptosis and autophagy in MOLT-4 and NB4 leukemic cell lines.

## Materials and Methods

### Preparation of sesame protein hydrolysate

Black sesame seeds were purchased from local supermarket (Nakhon Pathom, Thailand). To obtain protein hydrolysate, sesame seeds were ground and dissolved in 10 mM sodium acetate buffer pH 4.0 with pepsin hydrolysis at 37 °C for 18 h. Following the incubation period, the enzyme activity was stopped by boiling for 10 min. The protein hydrolysate in supernatant was collected by centrifugation at 5,000 rpm for 20 min, and stored at 4 °C for further purification.

### Determination of protein concentration by the Bradford assay

The protein concentration was determined using the Bradford assay (Bradford, 1976[2]). The bovine serum albumin was used for setting a standard curve.

### Purification of sesame peptides by reverse phase-solid phase extraction

The protein hydrolysate from sesame seeds was filtered through a 3 kDa cutoff membrane and fractionated by reverse phase-solid phase extraction (RP-SPE) using Sep-Pak C18 cartridge (Waters, Milford, MA, USA). The cartridge was pre-conditioned by acetonitrile and equilibrated by water. Then, the cartridge was loaded by sesame peptides and washed with water. The elution was done with 10 % stepwise concentration of acetonitrile at 1 ml/min of flow rate. Each 5 ml of eluates was pooled, removed acetonitrile using vacuum concentrator and determined the cytotoxicity in MOLT-4 and NB4 leukemic cell lines. The effective samples were selected for subsequent purification.

### Purification of sesame peptides by off-gel fractionation

The samples from RP-SPE that showed the cytotoxic effects were selected for purification by off-gel fractionation using an Agilent 3100 OFFGEL Fractionator (Agilent Technologies, Santa Clara, CA, USA). The immobilized pH gradient strips (Immobiline™ DryStrip pH 3-10 NL, 13 cm, GE Healthcare, Uppsala, Sweden) were rehydrated with 0.1 % IPG buffer (50 μl/well) for 1 h. The samples were diluted to final concentration of 0.05 % IPG and 200 μl of diluted sample was loaded in each well. The 12-well separations were focused for 16 kVh with a maximum current of 50 μA and power of 200 mW. After focusing, residue solution in each well was collected, measured the protein concentration, and determined the cytotoxic activities. The fractions with high cytotoxic activities were cleaned up by PureSpeed C18 Desalting Tips (Mettler Toledo, Columbus, OH, USA), dried using a vacuum centrifugation, and resuspended in 0.1 % formic acid for injecting into the mass spectrometer.

### Nano liquid chromatography-tandem mass spectrometry analysis and peptide identification

The samples were injected into the Ultimate 3000 LC System (Thermo Fisher Scientific, Inc., Waltham, MA, USA), which coupled with an impact II tandem mass spectrometer and a captive spray ion source on a nanocolumn PepSwift monolithic column 100 μm x 50 mm (Bruker Daltonik GmbH, Bremen, Germany). The separation was performed using a linear gradient from 10-70 % of 80 % acetonitrile in 0.1 % formic acid within 20 min. The raw data were analyzed by DeCyder MS 2.0 software (GE Healthcare, Piscataway, NJ, USA) using PepDetect module for peptide detection and quantitation. The analyzed data were searched against the NCBI protein database of *Sesamum indicum* L. (35,825 proteins) for peptide matching by Mascot software (Matrix Science, London, UK) with interrogation of taxonomy (*Sesamum indicum*), variation modification (oxidation) and peptide charge (1+, 2+ and 3+). The peptides with high ID score which indicates the similarity between analyzed peptides and databases were selected to synthesize by GenScript Biotech (Nanjing, China) for determining the anti-leukemic activities.

### Leukemic cell culture and isolation of peripheral blood mononuclear cell (PBMC)

The human acute T-lymphocytic leukemic cell line MOLT-4 and human acute promyelocytic leukemic cell line NB4 were purchased from Cell Line Service GmbH (Eppelheim, Germany). The cell lines were maintained in RPMI-1640 medium supplemented with 10 % FBS and 1 % penicillin-streptomycin (Thermo Fisher Scientific, Inc., Waltham, MA, USA) in a humidified incubator at 37 °C with 5 % CO_2_. PBMCs from healthy donors' blood were isolated using Lymphoprep™ (Axis-Shield PoC AS, Oslo, Norway). In brief, the blood was diluted with PBS at 1:1 ratio and gently layered on top of Lymphoprep™ solution. The separation was performed by centrifugation at 800 x g for 30 min with brake off. The PBMC layer was harvested and washed twice with medium. The isolated PBMCs were used for further experiment. This study was conformed to the standard set by the Declaration of Helsinki and approved by the committee for research, Faculty of Medicine Ramathibodi Hospital, Mahidol University (COA. MURA2019/679).

### Determination of cytotoxic effect by MTT assay

The leukemic cells and PBMCs were treated with various concentrations of sesame peptides, synthetic IM-7 and/or daunorubicin. After incubation time, 10 μl of the 5 mg/ml MTT [3-(4,5-dimethylthiazol-2-yl)-2,5-diphenyltetrazolium bromide] (Thermo Fisher Scientific, Inc., Waltham, MA, USA) was added to each well, followed by incubation for 4 h at 37 °C. The formazan crystal was dissolved in 100 μl of solubilizing solution (10 % SDS in 0.01 N HCl) overnight at 37 °C. The absorbance was determined using microplate reader at 570 nm. The half maximal inhibitory concentration (IC_50_) was calculated from linear regression and used for further experiment.

### Determination of apoptotic cells by flow cytometry

MOLT-4 and NB4 cell lines were treated with IM-7 at the IC_50_ value. Following 24 and 48 h of incubation, the cells were washed twice with cold PBS. The apoptotic cells were determined using the FITC Annexin V Apoptosis Detection kit (BD Biosciences, San Jose, CA, USA) by staining the cells with 5 μl Annexin V-FITC and 5 μl propidium iodide (PI) for 15 min in the dark and analyzed by FACS Canto II flow cytometer (BD Biosciences, San Jose, CA, USA) within 1 h.

### Measurement of LC3-II autophagic marker by flow cytometry

The leukemic cells were treated with IM-7 at the IC_50_ concentration for 24 and 48 h. The LC3-II level was measured using FlowCellect™ Autophagy LC3 Antibody-based Assay kit (Merck KGaA, Darmstadt, Germany) according to the manufacturer's instructions. Briefly, at 30 min before the end of incubation time, 10 μl of diluted reagent A was added followed by incubation for 30 min at 37 °C. Then, the cell pellet was resuspended in 100 μl of reagent B followed by immediate spinning. The cells were stained with anti-LC3/FITC antibody for 30 min in the dark and analyzed by flow cytometer. The LC3-II level was calculated from the mean fluorescence intensity (MFI) by the following equation: relative LC3-II level = MFI (Treatment)/MFI (Control).

### Reverse transcription-quantitative PCR (RT-qPCR) analysis

The leukemic cells were treated with IM-7 at the IC_50_ concentration for 24 h. Total RNA was extracted using GENEzol™ reagent (Geneaid biotech, New Taipei City, Taiwan) and 1 μg of total RNA was converted to cDNA using RevertAid First Strand cDNA Synthesis kit (Thermo Fisher Scientific, Inc., Waltham, MA, USA). qPCR was performed with the master mix containing 2 μl template, 0.5 μl of each primer and 10 μl Luna^®^ Universal qPCR Master Mix (New England Biolabs, Ipswich, MA, USA) at the final volume of 20 μl with Bio-Rad CFX96 touch™ real-time PCR detection system (Bio-Rad, Hercules, CA, USA). The amplification conditions were initial denaturation at 95 °C for 15 sec, followed by 40 cycles of denaturation at 95 °C for 15 sec and extension at 60 °C for 30 sec. The mRNA expression was analyzed using the mean C_q_ value and represented as 2^-∆∆Cq^ using GAPDH as an internal control. The primers used in this study were shown in Table 1(Tab. 1).

### Statistical analysis

The results are exhibited as mean ± SEM. For single variable comparisons, Student's t-test was used. For multiple variable comparisons, data were analyzed by one-way ANOVA followed by Dunnett's test using GraphPad Prism 6. The statistically significant difference was defined as P<0.05.

## Results

### Sesame peptides exhibit anti-leukemic activities

**Figure 1:**
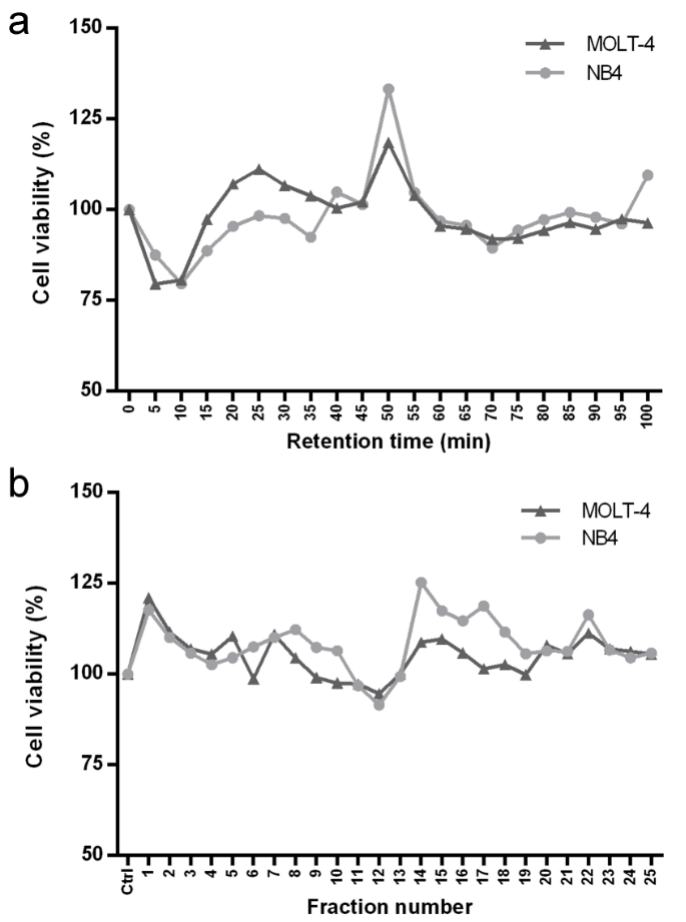
Cytotoxic effect of sesame peptides in MOLT-4 and NB4 cells. (a) The samples of sesame peptides obtained from RP-SPE were treated in MOLT-4 and NB4 cells at 35 μg/ml for 24 h followed by MTT assay. (b) The effective samples from RP-SPE were subsequently purified by off-gel fractionation. The obtained fractions were treated in MOLT-4 and NB4 cells at 25 μg/ml for 24 h followed by MTT assay.

After purification of sesame peptides by RP-SPE, each sample at 35 μg/ml was treated in MOLT-4 and NB4 cells for 24 h and the cytotoxicity was determined using an MTT assay. The results showed that the samples at retention time of 5, 10, 15 and 70 min had cytotoxic effects in both cell lines (Figure 1a(Fig. 1)). The effective samples from RP-SPE were further purified by off-gel fractionation. The 25 fractions obtained from off-gel fractionation (5, 8, 8 and 4 fractions from RP-SPE samples at retention time of 5, 10, 15 and 70 min, respectively) at 25 μg/ml were treated in MOLT-4 and NB4 cells for 24 h. The cytotoxic effects were examined by MTT assay. The results showed that fraction number 11 and 12 reduced the viability of MOLT-4 and NB4 (Figure 1b(Fig. 1)) and were subsequently selected to identify peptide sequences by nano LC-MS/MS.

### IM-7 exhibits the highest cytotoxicity in MOLT-4 and NB4 cells

**Figure 2:**
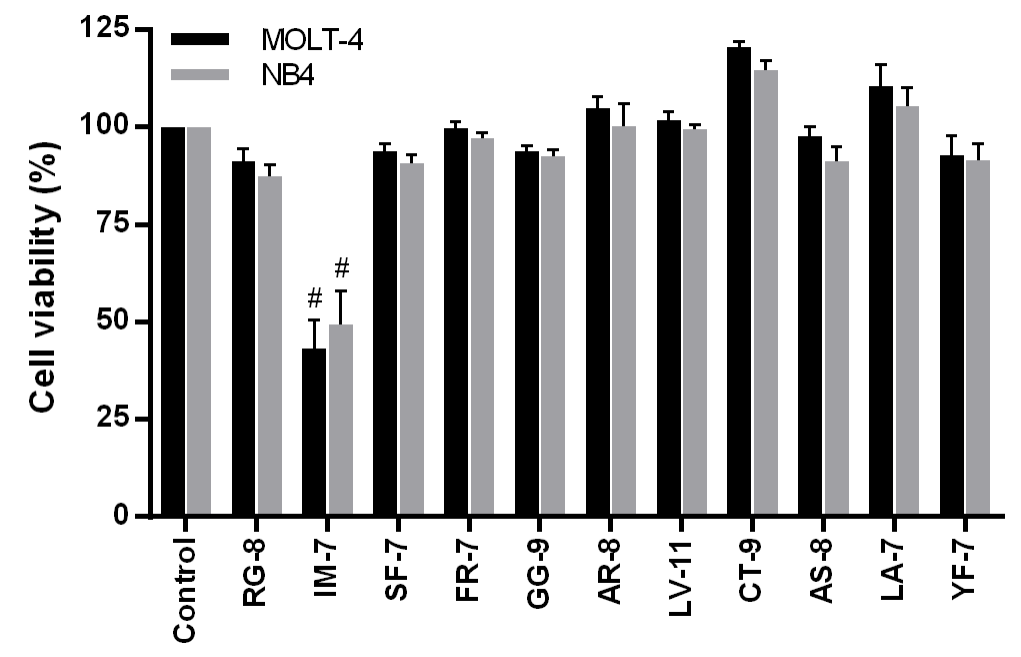
Screening for the cytotoxic effects of identified peptides in MOLT-4 and NB4 cells. MOLT-4 and NB4 cells were treated with eleven synthetic peptides obtained from DeCyder and Mascot analysis at 1 mg/ml for 24 h. The cell viability was determined by MTT assay. ^#^P<0.0001 vs. the control

The fraction number 11 and 12 obtained from the off-gel fractionation were injected into nano LC-MS/MS for peptide sequencing. The peptide sequences were analyzed by DeCyder MS 2.0 software and searched against the database of *Sesamum indicum* L. using Mascot software. The sequence of eleven peptides with high ID score and their located proteins were presented in Table 2(Tab. 2). The eleven peptides were synthesized with purity ≥ 85 % and screened for their cytotoxic effects in MOLT-4 and NB4 cells at 1 mg/ml for 24 h of incubation using an MTT assay. As shown in Figure 2(Fig. 2), IM-7 (IGTLILM) significantly reduced the proliferation of MOLT-4 and NB4 cells while other peptides did not show the cytotoxic effects. IM-7 was chosen for the further experiment.

### IM-7 selectively exhibits cytotoxicity to leukemic cells

**Figure 3:**
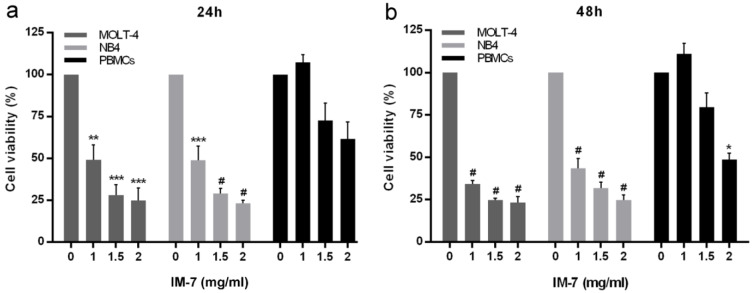
Effect of IM-7 in leukemic cells and healthy PBMCs. MOLT-4 and NB4 leukemic cells and healthy PBMCs were treated with various concentrations of IM-7 (0, 1, 1.5 and 2 mg/ml) for 24 h (a) and 48 h (b). The cell viability was determined using an MTT assay. ^*^P<0.05, ^**^P<0.01, ^***^P<0.001 and ^#^P<0.0001 vs. 0 mg/ml

The leukemic cells and PBMCs were treated with various concentrations of synthetic IM-7 (0, 1, 1.5 and 2 mg/ml) for 24 and 48 h. The cell viability was determined by MTT assay. The results revealed that IM-7 significantly inhibited the proliferation of MOLT-4 and NB4 in dose-dependent manner. The IC_50_ concentration of IM-7 at 24 h was 1.0 and 1.1 mg/ml in MOLT-4 and NB4, respectively. Remarkably, IM-7 had a minimal toxicity on healthy PBMCs at 1 and 1.5 mg/ml, but showed a significant effect at the highest concentration after 48 h of treatment (Figure 3(Fig. 3)).

### IM-7 induces apoptosis in MOLT-4 and NB4 cells

**Figure 4:**
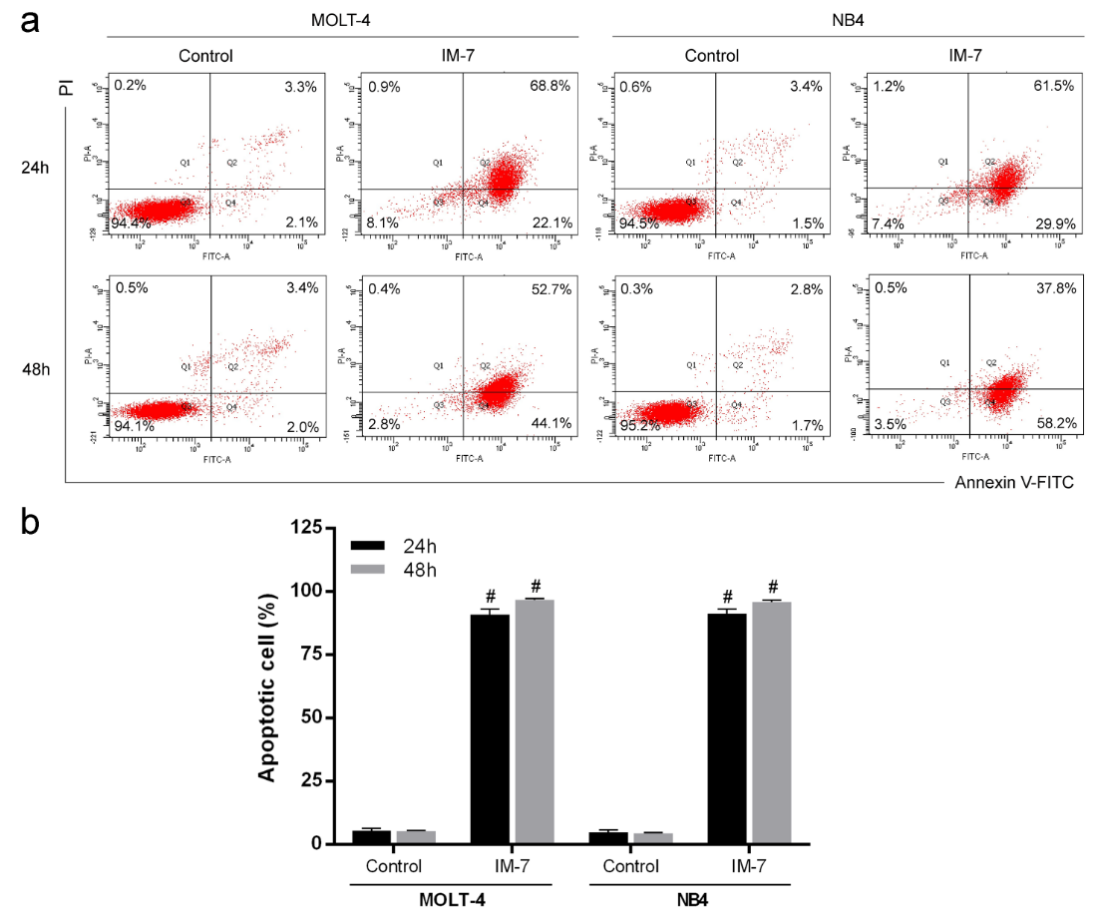
Effect of IM-7 on apoptosis in leukemic cells. (a) MOLT-4 and NB4 cells were treated with IM-7 at the IC_50_ value for 24 and 48 h. The apoptotic cells were analyzed by flow cytometry. (b) Quantified results of apoptosis assay. ^#^P<0.0001 vs. the control

To evaluate apoptosis induced by IM-7, MOLT-4 and NB4 cells were treated with IM-7 at the IC_50_ value. Following 24 and 48 h of incubation, the apoptotic cells were analyzed using Annexin V-FITC and PI staining followed by flow cytometry. The results showed that IM-7 induced apoptosis in MOLT-4 and NB4 by increasing the number of Annexin V-FITC-positive cells (Figure 4(Fig. 4)).

### IM-7 induces autophagy in MOLT-4 and NB4 cells

**Figure 5:**
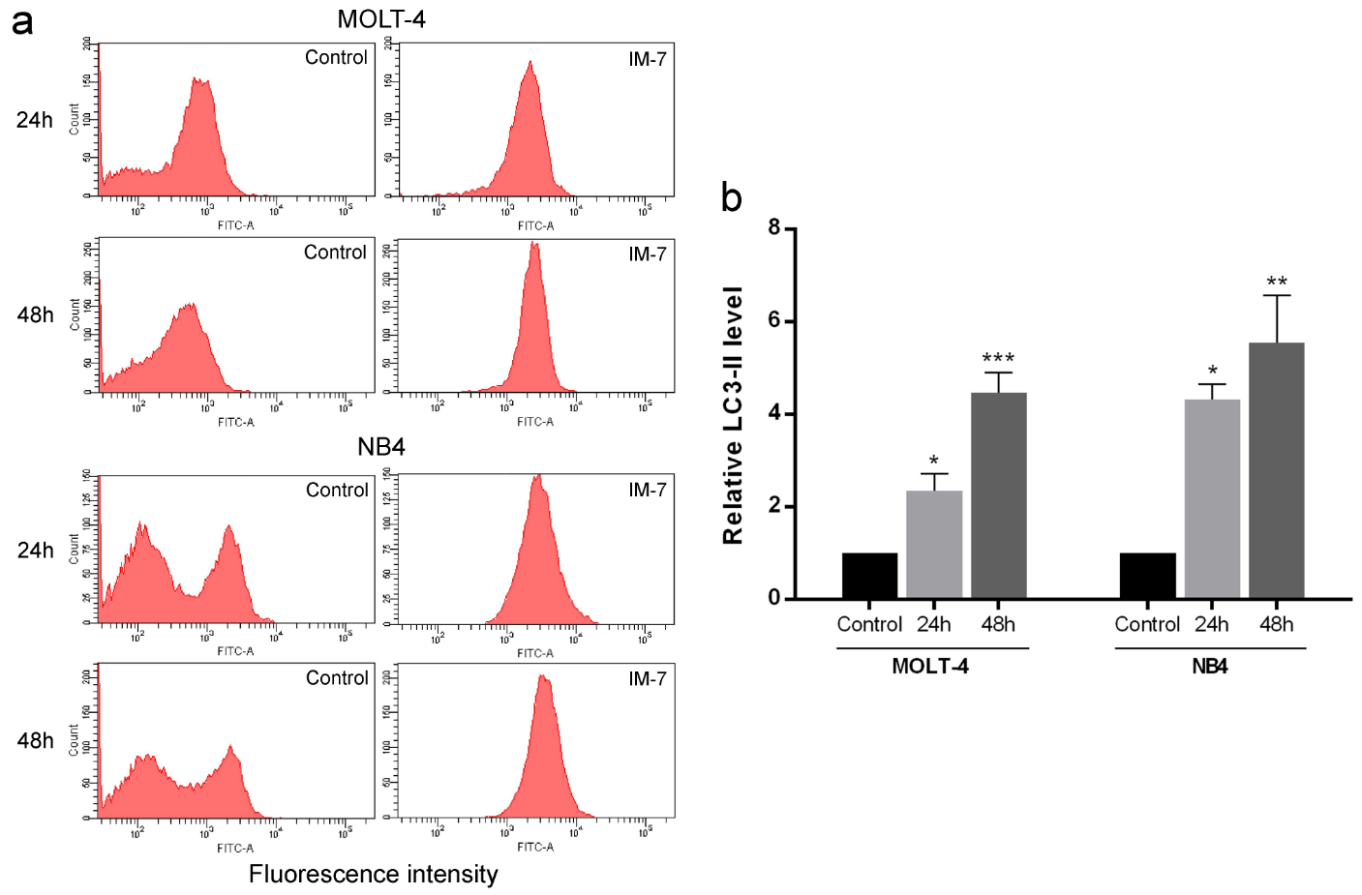
Effect of IM-7 on autophagy in leukemic cells. (a) MOLT-4 and NB4 cells were treated with IM-7 at the IC_50_ concentration for 24 and 48 h. The level of LC3-II autophagic marker was measured as mean fluorescence intensity using flow cytometry. (b) Quantified results of autophagy assay. ^*^P<0.05, ^**^P<0.01 and ^***^P<0.001 vs. the control

To investigate the induction of autophagy, LC3-II autophagic marker was measured. MOLT-4 and NB4 cells were incubated with IM-7 at the IC_50 _concentration for 24 and 48 h. The level of LC3-II was detected using anti-LC3/FITC antibody and flow cytometry. The results exhibited that IM-7 increased LC3-II level in MOLT-4 and NB4 in time-dependent manner compared to the control (Figure 5(Fig. 5)).

### IM-7 contributes to the alteration of apoptotic and autophagic mRNA expression

**Figure 6:**
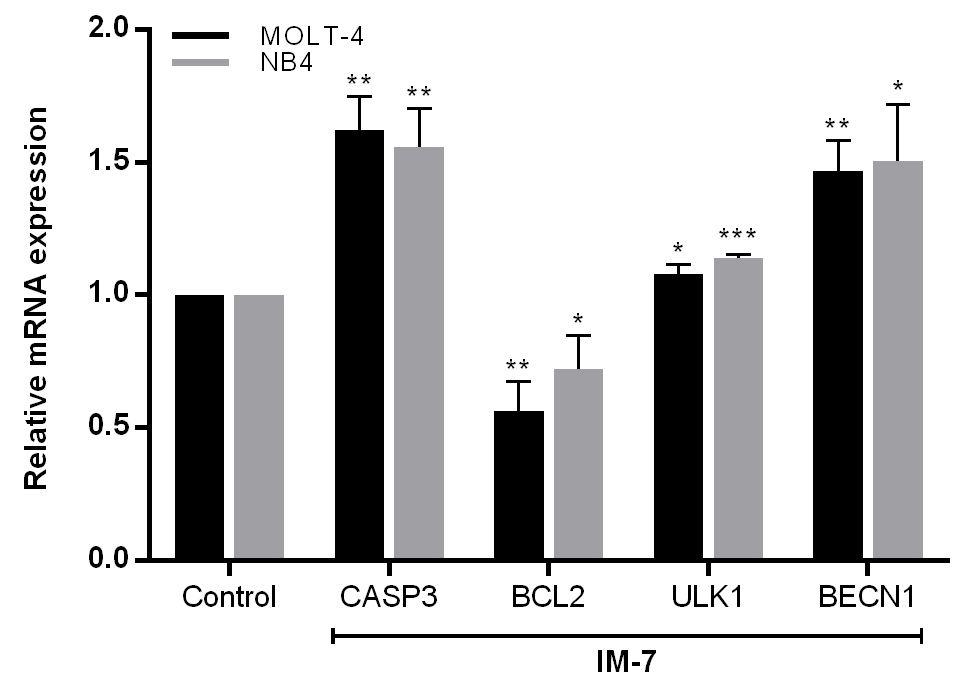
mRNA alteration of CASP3, BCL2, ULK1 and BECN1 in response of IM-7. MOLT-4 and NB4 cells were treated with IM-7 at the IC_50_ concentration for 24 h. The mRNA expression of CASP3, BCL2, ULK1 and BECN1 was assessed by RT-qPCR. ^*^P<0.05, ^**^P<0.01 and ^***^P<0.001 vs. the control

To assess the underlying mechanisms associated with apoptosis and autophagy, RT-qPCR was performed to analyze the mRNA expression of apoptotic genes, CASP3 and BCL2, and autophagic genes, ULK1 and BECN1. Compared to the control, IM-7 induced apoptosis through the upregulation of CASP3 and downregulation of BCL2, and induced autophagy through the upregulation of ULK1 and BECN1 (Figure 6(Fig. 6)).

### IM-7 enhances the cytotoxicity of anti-leukemic drug

**Figure 7:**
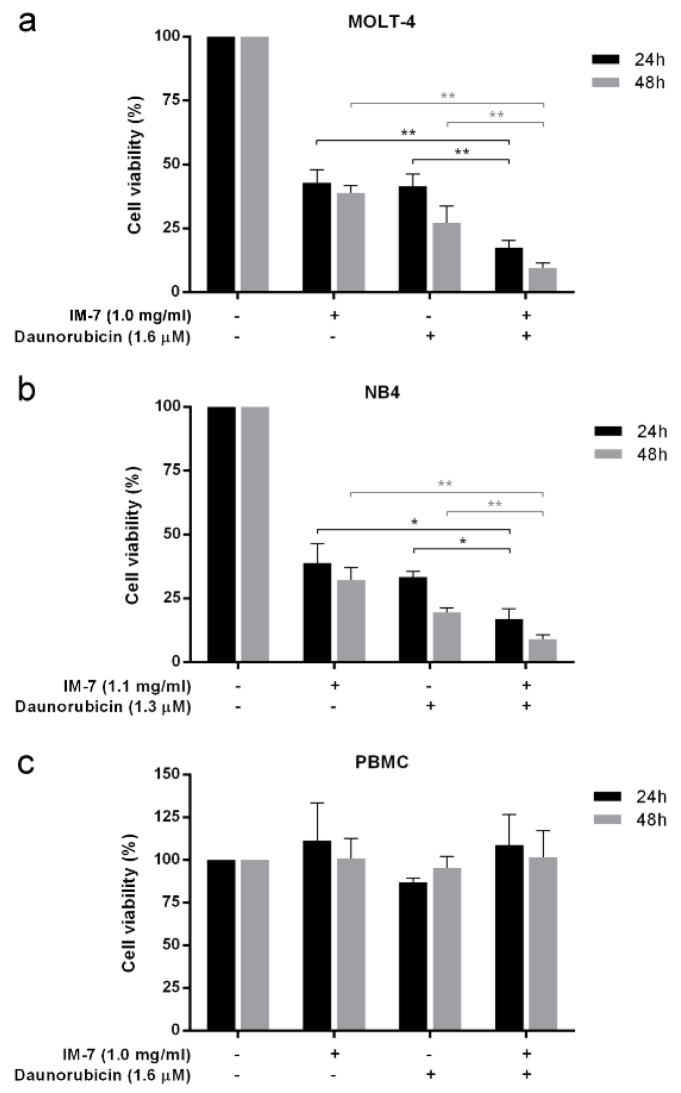
Effect of IM-7 in combination with daunorubicin. MOLT-4 (a) and NB4 (b) leukemic cells and healthy PBMCs (c) were treated with IM-7 at IC_50_ value with or without daunorubicin for 24 and 48 h. The cell viability was determined by MTT assay. ^*^P<0.05 and ^**^P<0.01.

To evaluate the role of IM-7 in complementary treatment, leukemic cells and PBMCs were treated with the combination between IC_50_ value of IM-7 (1.0 mg/ml for MOLT-4 and 1.1 mg/ml for NB4) and the well-known anti-leukemic drug daunorubicin (1.6 μM for MOLT-4 and 1.3 μM for NB4). Following 24 and 48 h of incubation, cell viability was determined by MTT assay. The results revealed that IM-7 enhanced the cytotoxicity of daunorubicin in MOLT-4 and NB4 compared to the single treatment of IM-7 or daunorubicin. However, this combination containing 1.0 mg/ml of IM-7 and 1.6 μM of daunorubicin appeared not to affect the cell viability of healthy PBMCs (Figure 7(Fig. 7)).

## Discussion

Over the past years, proteins and peptides have been developed for treatment in several diseases. More than 30 % of the therapeutic proteins and peptides database have been entered in clinical studies and 12 % of them have been approved as drugs by FDA (Lau and Dunn, 2018[18]). Among the FDA approved protein and peptide-based drugs, 12 % are classified as anticancer drugs (Usmani et al., 2017[31]) such as bortezomib for multiple myeloma (Chen et al., 2011[5]) and enfortumab vedotin-ejfv for bladder cancer (de la Torre and Albericio, 2020[8]). The present study discovered a bioactive peptide, namely IM-7 (IGTLILM), which was isolated from pepsin-treated protein hydrolysate of sesame seeds. It was found to possess anti-leukemic activities by inhibiting the proliferation of acute lymphocytic leukemia MOLT-4 and acute promyelocytic leukemia NB4 in dose-dependent manner. However, it exhibited less effect on healthy PBMCs. IM-7 majorly contained the hydrophobic amino acids that might be the important composition for cytotoxicity of leukemic cells. Previous studies have reported the positive correlation between anticancer activities and peptide hydrophobicity. The highly hydrophobic peptides can deeply penetrate into the hydrophobic core of cancer cell membrane followed by pore formation and cancer cell lysis (Huang et al., 2011[15]).

Apoptosis is the biological process of cell death characterized by cell shrinkage, membrane blebbing, phosphatidylserine exposure, chromatin condensation and DNA fragmentation. The mechanism is regulated by stimulation of pro-apoptotic factors and suppression of anti-apoptotic factors that are the promising strategies for development of the anticancer drugs (Pistritto et al., 2016)[26]. It was found that IM-7 induced the apoptosis of MOLT-4 and NB4 via the regulation of CASP3 and BCL2. Several studies have investigated the effect of natural-derived peptides on apoptosis in cancer cells. The peptides extracted from non-digestible fraction of the common beans have been shown to promote the apoptosis of colon cancer cells (Luna Vital et al., 2014). Lactoferricin, milk-derived peptide, has been shown to exert cytotoxic effect and increase apoptotic level in Jurkat leukemic cells (Mader et al., 2005[22]). Moreover, melittin, a polypeptide derived from the bee venom, has been found to induce apoptosis through the upregulation of caspase 3 and downregulation of Bcl-2 in U-937 leukemic cells (Moon et al., 2008[23]).

Autophagy is a cellular process characterized by the formation of autophagosomes and fusion with the lysosomes to degrade cellular components. It is regulated by several factors including autophagy-related genes (Atg), mammalian target of rapamycin (mTOR) and class III phosphatidylinositol 3-kinase (PtdIns3K) (Glick et al., 2010[12]). UNC-51-like kinase 1 (ULK1) and Beclin1 that play a vital role at initiated step of autophagy have been reported in the treatment of cancer. The activation of autophagy through AMPK/ULK1 by natural flavone, baicalein, was effective for the treatment of prostate and breast cancer (Aryal et al., 2014[1]). LYN-1604, a designed ULK1 agonist, has been shown to induce autophagic cell death through the activation of ULK1 in breast cancer cells (Zhang et al., 2017[35]). Moreover, Beclin1 has been reported as an insufficient tumor suppressor gene in several types of cancer and the disruption or deletion of Beclin1 can promote tumorigenesis (Qu et al., 2003[27]; Yue et al., 2003[34]). The results of the present study demonstrated that IM-7 activated autophagy by increasing the level of LC3-II in leukemic cells in time-dependent manner. The effect was supported by the upregulation of ULK1 and BECN1 expression.

In addition, the natural compounds have been proposed to improve the effects of anticancer drugs by synergizing the anti-proliferation activity, reducing the chemotherapy-induced toxicity and suppressing the development of drug resistance (Fu et al., 2018[10]; Kojima-Yuasa et al., 2015[17]; Rejhová et al., 2018[28]). In the present study, IM-7 enhanced the effect of anti-leukemic drug daunorubicin. However, this combination had a minimal effect on healthy PBMCs. This suggested that co-treatment of IM-7 and daunorubicin has a synergistic effect on anti-leukemic activities in MOLT-4 and NB4 cells.

Taken together, the present study demonstrated the anti-leukemic activities of IM-7 peptide isolated from the sesame hydrolysate. It was potent to inhibit the proliferation of leukemic cells, activate apoptosis and autophagy pathway, and synergize the sensitivity of anti-leukemic drug. The findings may be beneficial for the development of therapeutic peptides in human leukemia. The further investigations are essential to develop this peptide as an anti-leukemic agent.

## Acknowledgement

This work was supported by the Royal Golden Jubilee PhD scholarship (PHD/0051/2558 and PHD/0016/2560) from the Thailand Research Fund.

## Conflict of interest

No conflict of interest was reported by the authors.

## Figures and Tables

**Table 1:**
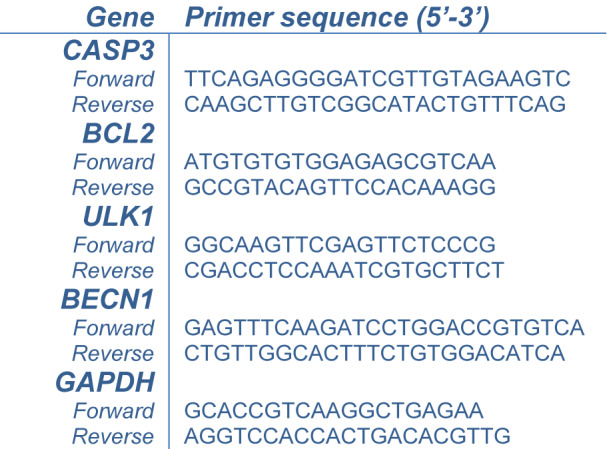
The primers for RT-qPCR analysis

**Table 2:**
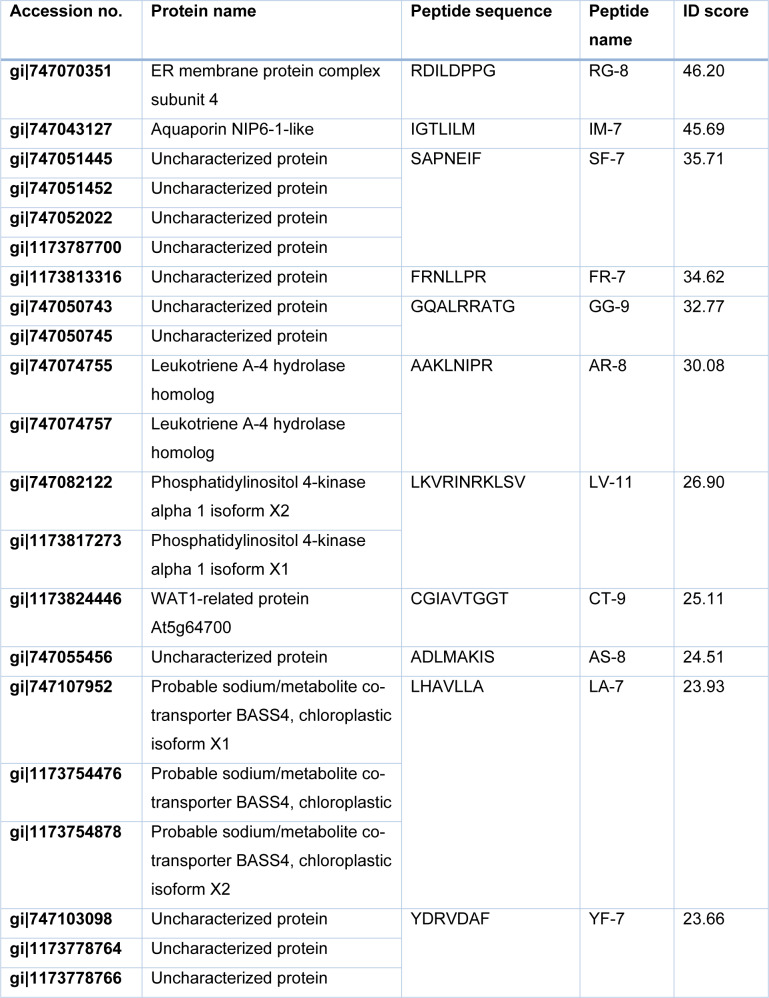
The identified sesame peptides and their located proteins
